# Histidine acid phosphatase domain-containing protein from *Haemonchus contortus* is a stimulatory antigen for the Th1 immune response of goat PBMCs

**DOI:** 10.1186/s13071-022-05411-7

**Published:** 2022-08-06

**Authors:** Zhaohai Wen, Zhaoying Zhang, Kalibixiati Aimulajiang, Muhammad Tahir Aleem, Jiajun Feng, Meng Liang, Mingmin Lu, Lixin Xu, Xiaokai Song, Xiangrui Li, Ruofeng Yan

**Affiliations:** 1 MOE Joint International Research Laboratory of Animal Health and Food Safety, College of Veterinary Medicine, Nanjing Agricultural University, Nanjing, 210095 Jiangsu People’s Republic of China; 2 State Key Laboratory of Pathogenesis, Prevention and Treatment of High Incidence Diseases in Central Asia, Xinjiang Medical University, Urumqi, 830011 Xinjiang People’s Republic of China

**Keywords:** *Haemonchus contortus*, Histidine acid phosphatase, Cytokines, PBMCs, Immunomodulation

## Abstract

**Background:**

Histidine acid phosphatase (HAP), a member of the histidine phosphatase superfamily, is widely found in parasites and is also a potential vaccine antigen or drug target. However, the biological function of HAP in *Haemonchus contortus* is still unclear.

**Methods:**

We cloned the HAP gene from *H. contortus* (Hc-HAP) and expressed the purified recombinant Hc-HAP (rHc-HAP) protein. The transcription of the Hc-HAP gene in the eggs, infective third-stage larvae (L3s), exsheathed third-stage larvae (xL3s) and adults (females/males) was analyzed by quantitative real-time-PCR (qPCR). An immunofluorescence assay was also used to detect the localization of Hc-HAP expression in adult worms. The effect of rHc-HAP on the function of peripheral blood mononuclear cells (PBMCs) was observed by co-culture of rHc-HAP protein with goat PBMCs.

**Results:**

The qPCR results revealed that the Hc-HAP gene was transcribed at a higher level in the L3 and xL3 stages that there were gender differences in transcription at the adult stage, with females exhibiting higher transcription than males. Moreover, Hc-HAP was mainly expressed in adult intestinal microvilli. Additionally, western blot results revealed that rHc-HAP could be detected in goat sera artificially infected with *H. contortus*. In the experiments, rHc-HAP bound to goat PBMCs and released nitric oxide. The rHc-HAP also induced the expression of interferon gamma (IFN-γ) and the phosphorylated STAT 1 transcription factor, while inhibiting interleukin-4 expression.

**Conclusions:**

The results shows that rHc-HAP stimulated the IFN-γ/STAT1 signaling pathway and enabled polarization of PBMCs toward T-helper 1 immune responses.

**Graphical Abstract:**

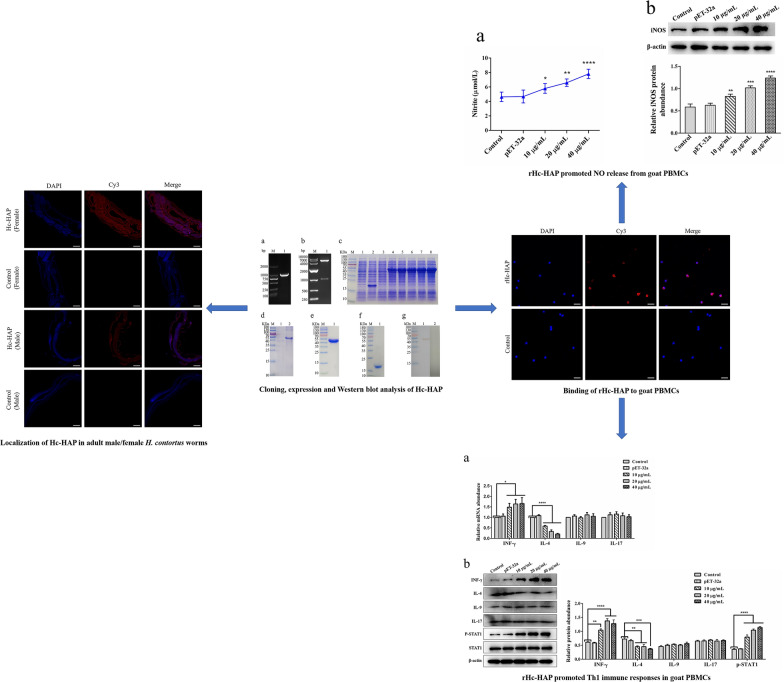

**Supplementary Information:**

The online version contains supplementary material available at 10.1186/s13071-022-05411-7.

## Background

*Haemonchus contortus* is one of the most debilitating parasites, inflicting enormous losses on the global livestock industry and negatively impacting animal welfare [1, 2]. In livestock, parasite control is currently accomplished using three major classes of anthelmintics: benzimidazoles, macrolides and imidazothiazoles [1, 3]. However, excessive use of anthelmintics in domestic animals can lead to nematode resistance [3, 4], and the emergence of multi-drug-resistant nematodes poses a potential threat to public health [5–10]. To develop an effective vaccine, it is imperative to understand the mechanisms of host-nematode interactions, particularly how nematode antigenic molecules modulate the host immune system.

The coexistence of *H. contortus* with the host relies on a delicate and complex immunomodulatory relationship between *H. contortus* and the host immune system. Gadahi et al. discovered that the excretory secretions of *H. contortus* inhibit the function of goat peripheral blood mononuclear cells (PBMCs) [11]. Moreover, we have recently demonstrated that trehalose-6-phosphate phosphatase (HcGOB) and trehalose phosphate synthase structural domain protein (Hc-TPS) in *H. contortus* inhibit PBMC function by activating the interleukin (IL)-10/signal transducer and activator of transcription (STAT) 3 (IL-10/STAT3) pathway [12, 13].

Histidine acid phosphatase (HAP), a member of the histidine phosphatase superfamily, is characterized by a conserved catalytic core (histidine centric) in which histidine itself is phosphorylated [14]. A member of the HAP superfamily is found in a wide variety of parasites, so inhibiting its function has a potential therapeutic benefit [14]. For example, the lysosomal acid phosphatase of *Giardia lamblia* is required for its excystation [15]. RNA interference (RNAi) silencing of *Caenorhabditis elegans* pho-1 revealed its maternal effects; in that study, Tetsunari et al. demonstrated that HAP is essential for the development and growth of the worm and that its loss is lethal to the worm [16]. However, the biological function of HAP in *H. contortus* remains to be elucidated.

It has been shown that acid phosphatase (homolog of HAP) isolated from the external surface of the intracellular parasite *Leishmania donovani* can inhibit the production of oxidative metabolites in neutrophils [17]. However, almost nothing is known about the effect of HAP in nematodes on host immune function.

As part of the present study, we characterized the transcriptional features of the HAP gene from *H. contortus* (Hc-HAP) in the *H. contortus* egg, infective third-stage larvae (L3s), exsheathed third-stage larvae (xL3s) and adults, as well as its expression patterns in female and male adults. We also assessed the effects of the purified recombinant Hc-HAP (rHc-HAP) protein on immune cell function in goats, which may be helpful in developing new vaccine targets.

## Methods

### Parasites, animals and PBMCs

Native goats (3–6 months old) were housed in the Laboratory Animal Center at the School of Animal Medicine and provided with sterile hay, corn feed and free access to water. *Haemonchus contortus* (Nanjing strain) were maintained by successive passages through worm-free local hybrid goats. Adults (females and males), L3s, eggs and xL3s were collected as described in previous studies [11, 18]. Healthy goats were housed in individual cages to prevent accidental worm infections and provided with sterile hay, corn feed and free access to water.

Wistar rats weighing approximately 250 g were purchased from Qing Long Shan Animal Breeding Farm, housed in a barrier animal house at the Experimental Animal Center of Nanjing Agricultural University and provided with sterile food and water.

PBMCs were isolated from goat peripheral blood using the standard Ficoll-Hypaque (GE Healthcare, Little Chalfont, UK) gradient centrifugation method described previously [19]. The PBMCs were washed 3 times with sterile phosphate-buffered solution (PBS), and the cell density was adjusted to 1 × 10^6^ cells/ml. Then, the cell viability of isolated PBMCs was detected with trypan blue dye [12].

### Bioinformatics and molecular modeling

Nucleotide sequence and amino acid sequence alignment analysis were performed using the Protein BLAST program on the NCBI website. The amino acid sequences of Hc-HAP were aligned with homologous sequences of other species, namely *Teladorsagia circumcincta* (NCBI: PIO70295.1), *Ancylostoma ceylanicum* (NCBI: EPB66915.1), *Necator americanus* (NCBI: XP_013292873.1), *Caenorhabditis briggsae* (NCBI: XP_002630680.1), *Caenorhabditis remanei* (NCBI: XP_003108969.1), *Caenorhabditis elegans* (NCBI: NP_494983.1), *Oesophagostomum dentatum* (NCBI: KHJ94763.1), *Teladorsagia circumcincta* (NCBI: PIO64973.1), *Loa loa* (NCBI: XP_003136999.1) and *Brugia malayi* (NCBI: XP_001896424.1). In addition, the spatial structure of the Hc-HAP protein was predicted by using the SWISS-MODEL server homology modeling program (https://swissmodel.expasy.org/interactive). The SignalP 5.0 Server (http://www.cbs.dtu.dk/services/SignalP/) and TMHMM Server v. 2.0 (http://www.cbs.dtu.dk/services/TMHMM/) were used to predict signal peptides and transmembrane regions, respectively.

### Construction of recombinant expression plasmids and expression of the rHc-HAP protein

*Haemonchus contortus* adults were obtained from the fourth stomach of goats infected with *H. contortus*. As a first step, total RNA of adult worms was isolated by TRIzol reagent (Vazyme Biotech Co., Ltd., Nanjing, China). Then, 1 µg of RNA was synthesized into complementary DNA (cDNA) using the HiScript III First Strand cDNA Synthesis Kit (Vazyme Biotech Co., Ltd.). Specific amplification primers (HAP-F and HAP-R; Additional file 1: Table S1) were synthesized based on the sequence of the coding region (CDS region) of the Hc-HAP gene (GenBank: CDJ80664.1) published on the NCBI website. A 25-µl aliquot of the reaction system [1 µl of each primer, 12.5 µl of 2× Phanta Master Mix (Vazyme Biotech Co., Ltd.), 1 µl of cDNA and 9. 5 µl of ddH_2_O] was prepared in a PCR tube and amplified by following the PCR procedure described by Wen et al. [13]. The gene products were cleaned using a Gel Extraction Kit (Vazyme Biotech Co., Ltd.) and ligated to the pET32a(+) prokaryotic expression vector. The pET32a(+)/Hc-HAP plasmid was verified by enzymatic digestion reactions (*Bam*HI/*Xho*I) and sequence alignment analysis with BLAST.

The rHc-HAP protein was expressed and purified as previously described [13]. The pET32a(+)/Hc-HAP plasmid was transformed into *Escherichia coli* BL21(DE3) and induced to express rHc-HAP protein using isopropyl-D-thiogalactoside (IPTG). The rHc-HAP protein was purified by Ni^2+^-nitrilotriacetic acid (Ni–NTA) columns according to the manufacturer's instructions. Then, the rHc-HAP protein was refolded by sequential passage through buffers (500 mmol/l NaCl, 20 mmol/l Tris–HCl, 0.1 mmol/l GSSG, 1 mmol/l GSH) containing a decreasing concentration of urea (8, 6, 4, 3, 2, 1 and 0 M). Lipopolysaccharide was removed from rHc-HAP protein using the ToxinEraserTM Endotoxin Removal kit (GenScript, Nanjing, China). Finally, 12% sodium dodecyl sulfate-polyacrylamide gel electrophoresis (SDS-PAGE) was used to detect the purification effect of rHc-HAP protein.

### Preparation of polyclonal antibodies against rHc-HAP protein

The rat anti-Hc-HAP polyclonal antibody was prepared as described previously [13]. Briefly, rHc-HAP protein (300 μg) was mixed with adjuvant (Freund's complete adjuvant; Sigma-Aldrich, St. Louis, MO, USA) and immunized subcutaneously in Wistar rats. After 14 days, rHc-HAP protein (300 μg) was mixed with adjuvant (Freund’s incomplete adjuvant; Sigma-Aldrich) and injected subcutaneously at multiple points in Wistar rats, followed by the injection of three more doses of rHc-HAP protein (300 μg) mixed with Freund’s incomplete adjuvant at 7-day intervals. One week after the fifth immunization, antisera to rHc-HAP protein were collected and stored at − 80 °C.

### Analysis of rHc-HAP protein by western blot assays

The relative protein expression of rHc-HAP was detected by western blot, as described in our previous studies [12, 20]. Briefly, the proteins (30 µg) were transferred onto polyvinyl difluoride (PVDF) membranes [GE Healthcare Life Science (China) Co., Beijing, China] after 12% SDS-PAGE and the membranes incubated with 5% bovine serum albumin (BSA) at 37 °C for 1 h. After washing the membranes 5 times with TBST (Tris-buffered saline containing 0.05% Tween-20), the membranes were incubated overnight at 4 °C with goat serum artificially infected with *H. contortus*. The following day, the membranes were washed 5 times with TBST and incubated with rabbit anti-goat immunoglobulin G (H+L) horseradish peroxidase secondary antibody for 2 h at room temperature. Finally, the bound antibodies were detected following the instructions of the DAB Horseradish Peroxidase Chromogenic Kit (Beyotime, Shanghai, China).

### Detection of the relative transcript levels of the Hc-HAP gene using quantitative real-time PCR

Detection of relative transcript levels of Hc-HAP genes in different life stages (adults, L3s, eggs and xL3s) was performed by quantitative real-time PCR (qPCR) using primers (Additional file 1: Table S2) as previously described [12, 21]. Briefly, total RNA was isolated from adults (female and male), eggs, L3s and xL3s by the TRIzol method (Vazyme Biotech Co., Ltd.). Then, 1 µg of RNA was synthesized into cDNA using the HiScript III First Strand cDNA Synthesis Kit (Vazyme Biotech Co., Ltd.). The β-tubulin gene of *H. contortus* was used as a reference gene. The data were analyzed according to raw cycle thresholds (Ct), which were obtained using ABI Prism 7500 software (Applied Biosystems, Foster City, CA, USA) using the comparative Ct (2^−ΔΔ Ct^) method.

### Immunolocalization of Hc-HAP

The expression of native Hc-HAP protein in adult worms (females and males) was detected by immunofluorescence assay (IFA). *Haemonchus contortu*s adults were obtained from the fourth stomach of goats and fixed in 4% tissue cell fixation solution for 12 h as previously described [12]. Samples were prepared into 4-μm paraffin sections, and antigens were repaired by microwave heating. Sections were placed in 5% BSA solution and incubated for 1 h at 37 °C, followed by incubation with rat anti-rHc-HAP serum and normal rat serum (negative control) overnight at 4 °C. The following day, after five washes with PBST wash solution, the sections were incubated with Cy3-labeled Goat Anti-Rat IgG (Beyotime, Shanghai, China) for 1 h at 37 °C, while being protected from light. After 5 washes with PBST, sections were stained with 4′,6-diamidino-2-phenylindole (DAPI; Beyotime), and fluorescence was observed with a laser confocal microscope (LSM 710; Carl Zeiss Spectroscopy GmbH, Jena, Germany).

### Binding of rHc-HAP protein to goat PBMCs

The ability of the rHc-HAP protein to bind to goat PBMCs was examined following procedures described previously [12]. PBMCs were incubated with rHc-HAP protein (10 μg/ml) at 37 °C for 1 h in a 5% CO_2_ cell culture incubator, and a control group was established (an equal volume of PBS was added). The PBMCs were washed with PBS and transferred to slides, following which the slides were fixed with 4% tissue cell fixation solution for 20 min. The specific IFA procedure was the same as that used for the immunolocalization assay of Hc-HAP.

### Cell proliferation assays

Cell proliferation was detected using the Cell Counting Kit-8 (CCK-8; Beyotime) according to our previous report [13]. Briefly, freshly isolated PBMCs were spread on 96-well cell culture plates at a concentration of 1 × 10^6^/ml with 100 µl per well. Different concentrations (10, 20, 40 μg/ml) of rHc-HAP protein or pET-32a protein were added, respectively, and a control group was established (an equal volume of PBS was added). The cell culture plates were incubated in a 5% CO_2_ cell incubator at 37 °C for 24 h. Following the addition of 10 μl of CCK-8 solution to each well, the cells were incubated for a further 4 h, at which time point the absorbance values at 450 nm were detected using a miniature flat panel reader.

### Cell apoptosis assays

The group settings were the same as those used for the cell proliferation assays. The effect of rHc-HAP protein on the apoptosis of PBMCs was detected using flow cytometry, as previously described [22]. Cells were stained using the Annexin V-FITC Apoptosis Detection Kit (Beyotime) according to the manufacturer's instructions. The stained cells were analyzed by flow cytometry.

### Nitric oxide production assays

The group settings were the same as those used for the cell proliferation assays. The cells were incubated in a 5% CO_2_ cell incubator for 24 h at 37 °C, and the supernatant was collected. The secreted levels of nitric oxide (NO) in different groups were detected by the Total Nitric Oxide Assay Kit (Beyotime).

### Cytokine transcript abundance detected by qPCR

The group settings were the same as those used for the cell proliferation assays. The relative transcript abundance of IL-4, IL-9, IL-17, and interferon gamma (IFN-γ) was analyzed by qPCR. The reaction system contained 5 μl 2× ChamQ SYBR qPCR Master Mix (Vazyme Biotech Co., Ltd.), 3.6 μl ddH_2_O, 0.2 μl forward and reverse primers and 1 μl cDNA. Primer sequences are shown in Additional file 1: Table S2 [23, 24]. To calculate raw cycle thresholds (Ct), the relative Ct (2^−ΔΔCt^) method was used with ABI Prism 7500 software (Applied Biosystems).

### The expression levels of inducible nitric oxide synthase, IL-17, IL-4, IFN-γ, IL-9 and p701-STAT1 were detected by western blot assays

The group settings were the same as those used for the cell proliferation assays. The specific procedure was performed as described previously for western blot assays in section Analysis of rHc-HAP protein by western blot assays. The expression of the target proteins was detected by chemiluminescence. Image J software was used to analyze the relative protein expression levels. The source antibodies were: inducible nitric oxide synthase (iNOS), IL-4, IFN-γ, IL-17, STAT1 and p701-STAT1 (1:1000; Affinity Biosciences, China); IL-9 (prepared by our research team); and β-actin (ABclonal, Wuhan, China).

### Data analysis

Student’s t-test and one-way analysis of variance (ANOVA) analyses were used to compare two groups and different groups, respectively. Data are presented as the mean ± standard error of the mean and were considered significant at *P < 0.05, **P < 0.01, ***P < 0.001 and ****P < 0.0001. All experiments were repeated a minimum of three times.

## Results

### Cloning, expression and western blot analysis of Hc-HAP

**Fig. 1:**
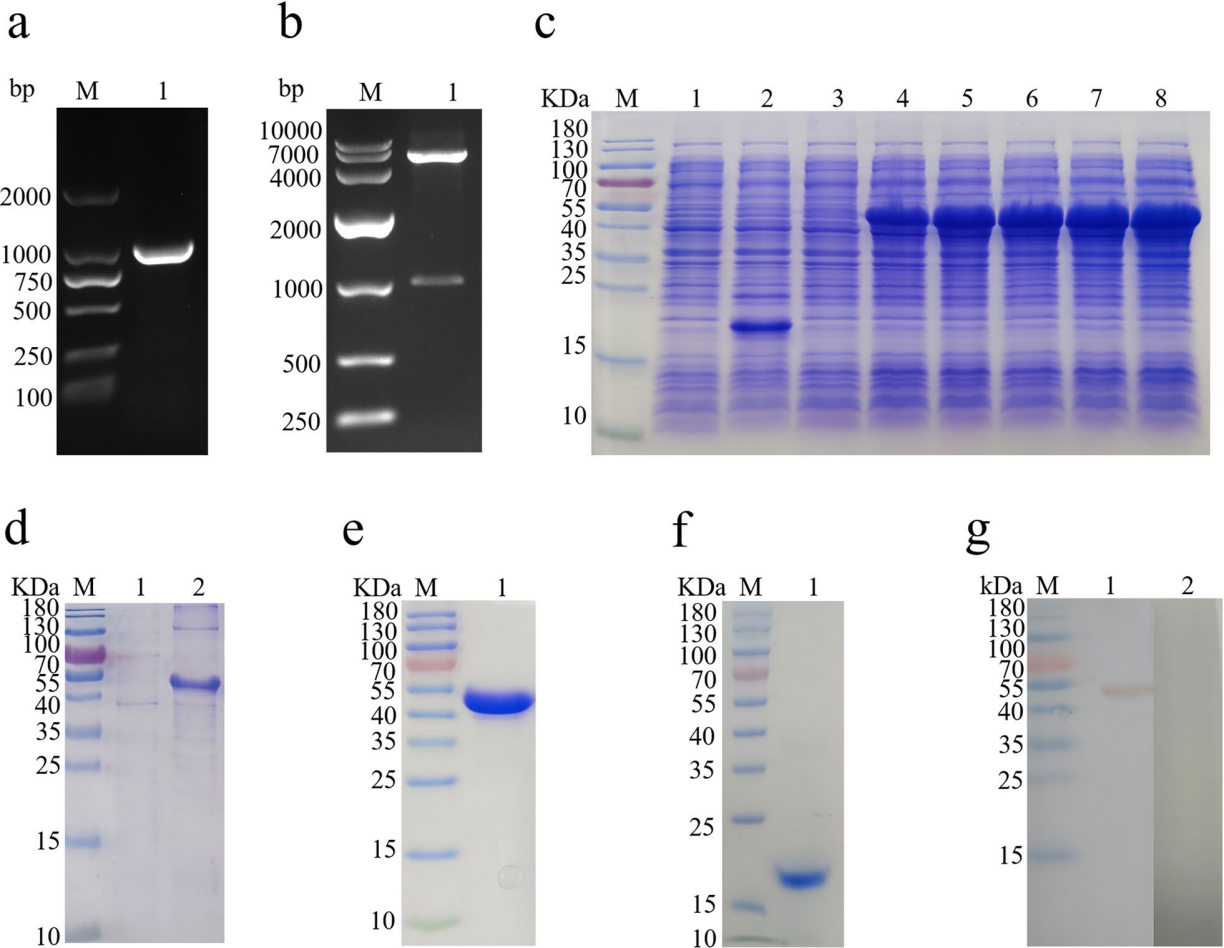
Cloning, expression and western blot analysis of Hc-HAP. **a** Amplification of Hc-HAP. Lanes:* M* DNA marker DL 2000,* 1* amplification products of the Hc-HAP gene. **b** Lanes:* M* DNA marker DL10000,* 1* digestion of pET-32a/Hc-HAP plasmid by enzymes. **c**–**f** Expression and purification of rHc-HAP and pET-32a. Lane:* M* Standard protein molecular weight marker. **c** Lanes:* 1*,* 2* pET-32a induced by IPTG for 0 and 5 h,* 3–8* pET-32a/Hc-HAP induced by IPTG for 0 to approx. 5 h. **d** Lanes:* 1* Supernatant of expression products,* 2* inclusion body of expression products. **e** Lane:* 1* purification of rHc-HAP. **f** Lane:* 1* purification of pET-32a. **g** Western blot analysis of rHc-HAP protein. Lanes:* M* Standard protein molecular weight marker,* 1* rHc-HAP detected by serum incubated with positive *H. contortus* goat,* 2* no protein was detected with normal goat sera. Abbreviations: Hc-HAP, histidine acid phosphatase gene from *H. contortus*; IPTG, isopropyl-β-D-thiogalactopyranoside; rHC-HAP, purified recombinant Hc-HAP

No signal peptide and transmembrane structure were found in the Hc-HAP protein sequence (Additional file 2: Figures S1, S2). The cDNA of adult *H. contortus* worms was used as a template to amplify the Hc-HAP gene using primers HAP-F and HAP-R, and the size of the amplified target gene was 990 bp (Fig. 1a). The Hc-HAP gene was successfully inserted into the pET32a vector and verified by enzymatic digestion and online BLAST analysis (Fig. 1b). The recombinant plasmid (pET32a/Hc-HAP) was induced by IPTG to be expressed in *E. coli* BL21 (DE3). After separation by SDS-PAGE, staining with Coomassie Brilliant Blue revealed that the size of the fusion protein rHc-HAP was approximately 52 kDa (Fig. 1c). The rHc-HAP protein was expressed in the form of inclusion bodies (Fig. 1d) and purified by passage through the HisTrap TM FF Column. SDS-PAGE showed a single band for the purified rHc-HAP, indicating good purification (Fig. 1e).

As shown in Fig. 1g, rHc-HAP was recognized by sera from goats infected with *H. contortus*, whereas normal goat sera did not recognize rHc-HAP. This result suggests that Hc-HAP is exposed to the host immune system during *H. contortus* infection and has the potential to be a vaccine candidate antigen.

### Sequence alignment analysis and molecular modeling of Hc-HAP

**Fig. 2:**
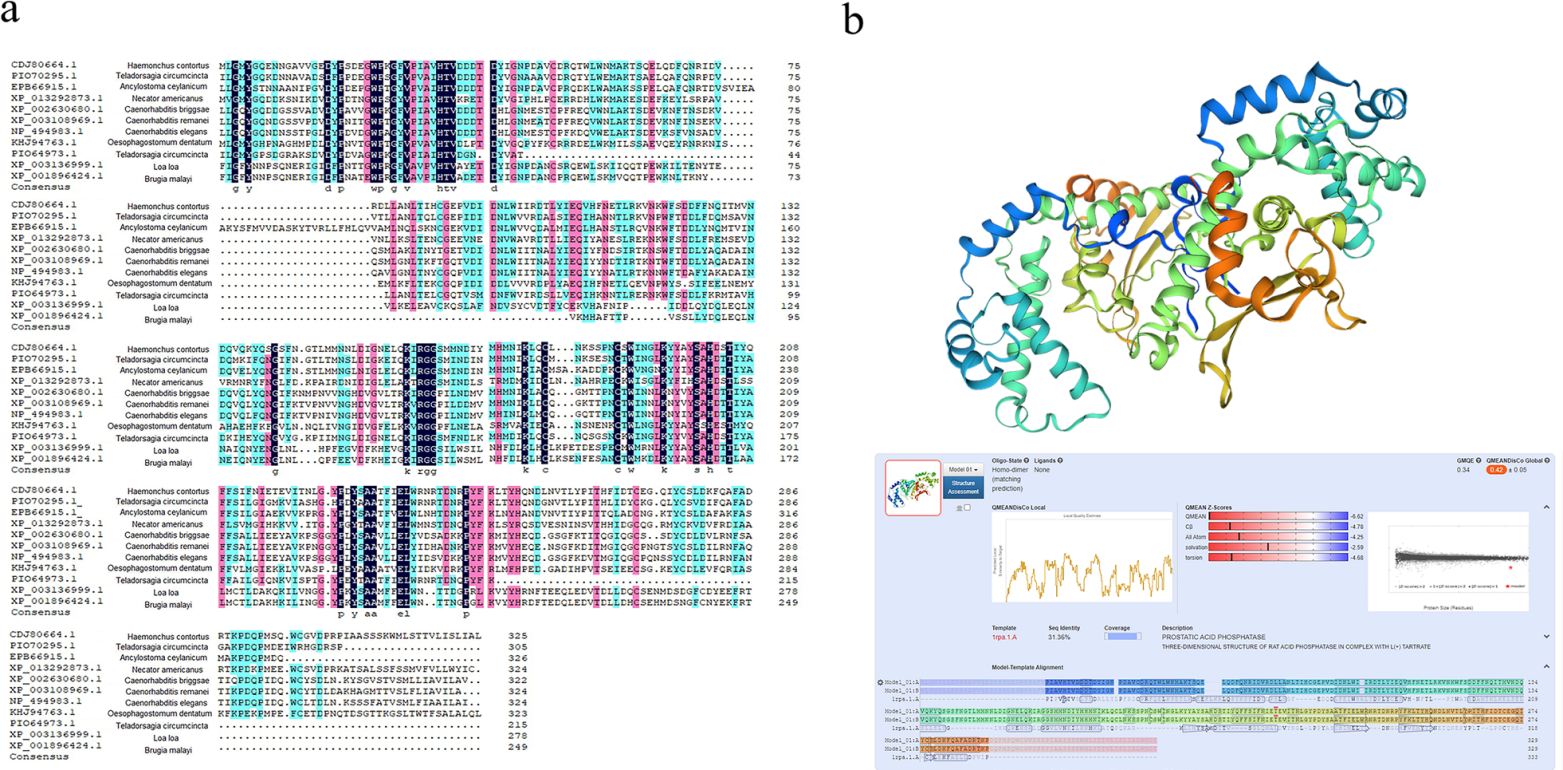
Sequence alignment analysis and molecular modeling of Hc-HAP. **a** Multiple sequence alignment analysis of amino acid sequences of Hc-HAP with HAP homologs from other species [*Teladorsagia circumcincta* (72.46%), *Ancylostoma ceylanicum* (57.67%), *Necator americanus* (48.48%), *Caenorhabditis briggsae* (48.77%), *Caenorhabditis remanei* (49.24%), *Caenorhabditis elegans* (47.26%), *Oesophagostomum dentatum* (42.81%), *Teladorsagia circumcincta* (55.65%), *Loa loa* (35.0%), *Brugia malayi* (32.41%)]. **b** The SWISSMODEL server provided the best template to predict the Hc-HAP three-dimensional model and acid phosphatase from rat (1rpa.1. A) with 31.36% identity

The cloned Hc-HAP gene sequence was confirmed by BLAST analysis, and the nucleotide sequence was translated into 329 amino acid residues by the ExPASy Translate tool (https://web.expasy.org/translate/). Multiple sequence alignment of Hc-HAP with available homologous sequences on the NCBI database revealed that Hc-HAP was closely related to the HAP of *Teladorsagia circumcincta* (72.46%), *Ancylostoma ceylanicum* (57.67%), *Necator americanus* (48.48%), *Caenorhabditis briggsae* (48.77%), *Caenorhabditis remanei* (49.24%), *Caenorhabditis elegans* (47.26%), *Oesophagostomum dentatum* (42.81%), *Teladorsagia circumcincta* (55.65%), *Loa loa* (35.0%) and *Brugia malayi* (32.41%) (Fig. 2a). The SWISS-MODEL server provided the best template to predict the Hc-HAP three-dimensional model and acid phosphatase from rat (1rpa.1. A) with 31.36% identity (Fig. 2b).

### Relative transcript abundance of Hc-HAP gene in different life stages of *H. contortus*

**Fig. 3:**
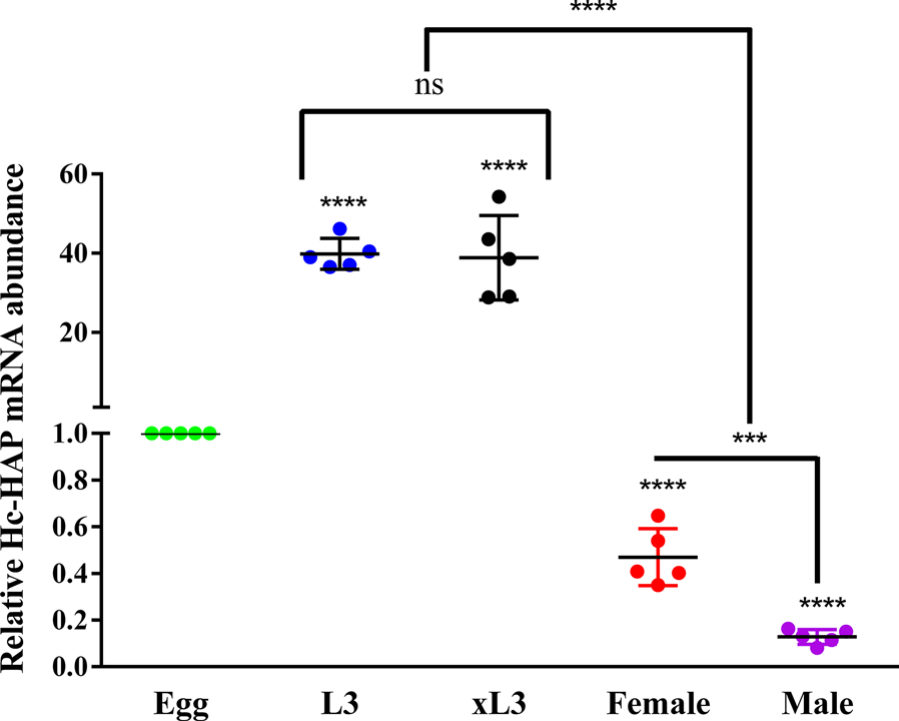
Transcriptional analysis of the Hc-HAP gene in different developmental stages of *H. contortus*. The relative quantities (compared with the egg state: Egg = 1) are shown as mean values ± SEM. The results presented here are representative of three independent experiments. Asterisks indicate significant differences at *P < 0.05, **P < 0.01, ***P < 0.001 and ****P < 0.0001. Abbreviations: L3, Infective third-state larva; mRNA, messenger RNA; ns, no significant difference; SEM, standard error of the mean; xL3s, exsheathed L3s

The relative transcript levels of the Hc-HAP gene in different developmental stages of the *H. contortus* were examined using qPCR. As shown in Fig. 3, Hc-HAP transcript levels were significantly upregulated at the L3 [t-test, *t*_(8)_ = 22.29, *P* < 0.0001] and xL3 [t-test, *t*_(8)_ = 7.916, *P* = 0.0014] stages compared to the egg stage. However, transcription was significantly downregulated in the adult [female (t-test, *t*_(8)_ = 9.737, *P* = 0.0006] and male [t-test, *t*_(8)_ = 60.91, *P* < 0.0001) stages and was higher in female adults than in male adults [t-test, *t*_(8)_ = 6.466, *P* = 0.0029].

### Immunolocalization of native Hc-HAP protein in adult *H. contortus*

**Fig. 4:**
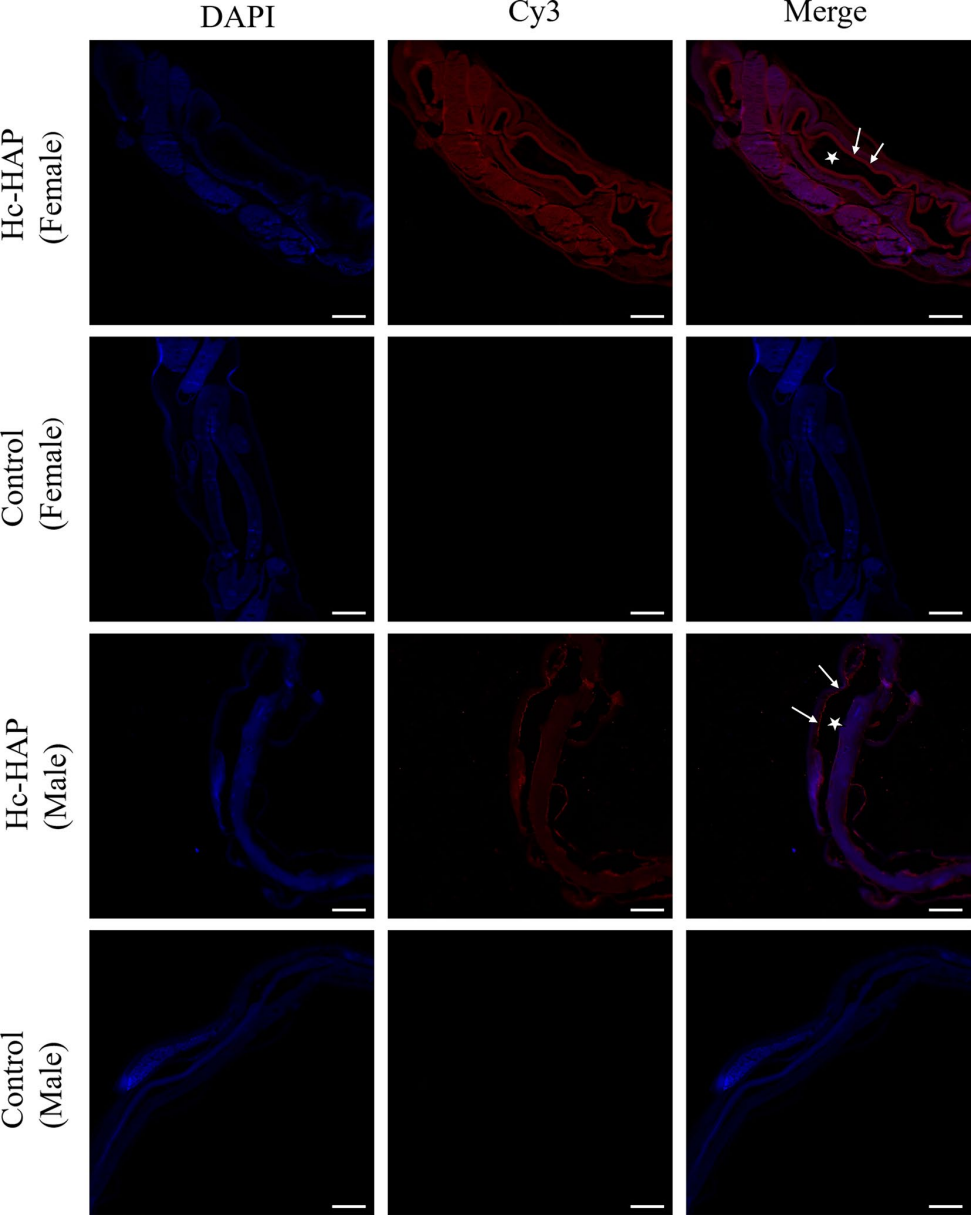
Immunolocalization of Hc-HAP in adult *H. contortus*. The red color indicates the localization of target protein stained with the Cy3 dye in adult worms, and the blue color indicates the localization of nuclei stained with the fluorescent stain DAPI. Merge image of DAPI and Cy3. Organs annotated in the Merge image are intestinal microvilli (white arrow), cavity of the intestine (asterisks). Scale bars: 100 μm

Localization of Hc-HAP protein in adult nematodes was examined by IFA. Figure 4 shows the longitudinal sections of female and male worms, respectively, where the red fluorescent signal represents the Hc-HAP protein. Expression of rHc-HAP was observed in the intestinal microvilli (the main expression sites of Hc-HAP protein), gonads and body wall of *H. contortus*. Control sections exhibited no red fluorescent signal.

### Binding of rHc-HAP protein to goat PBMCs

**Fig. 5:**
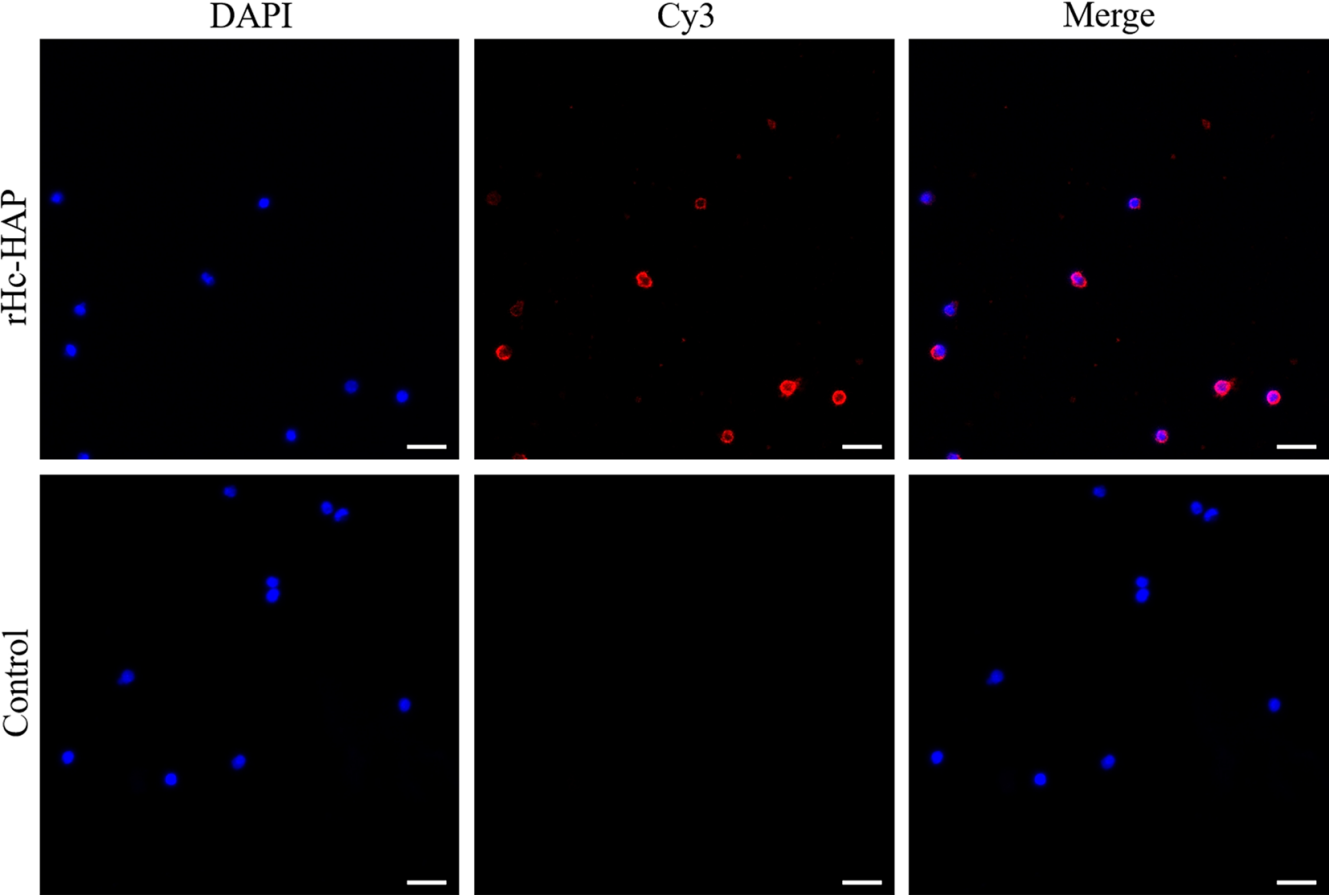
Binding of rHc-HAP protein to goat PBMCs. The red color indicates the localization of target protein stained with Cy3 on PBMCs and the blue color indicates the localization of nuclei stained with DAPI. Merge image of DAPI and Cy3. No red fluorescence was observed in the control. Scale bars: 20 μm. Abbreviation: PBMCs, Peripheral blood mononuclear cells

Goat PBMCs were incubated with rHc-HAP protein, and the ability of the PBMCs to bind to rHc-HAP protein was determined by IFA. The IFA results showed that red fluorescence was observed on the surface of PBMCs treated with rHc-HAP protein, while no red fluorescence was detected in the control group (Fig. 5). These results suggest that rHc-HAP protein can bind to goat PBMCs.

### Effect of rHc-HAP protein on the proliferation of goat PBMCs

**Fig. 6:**
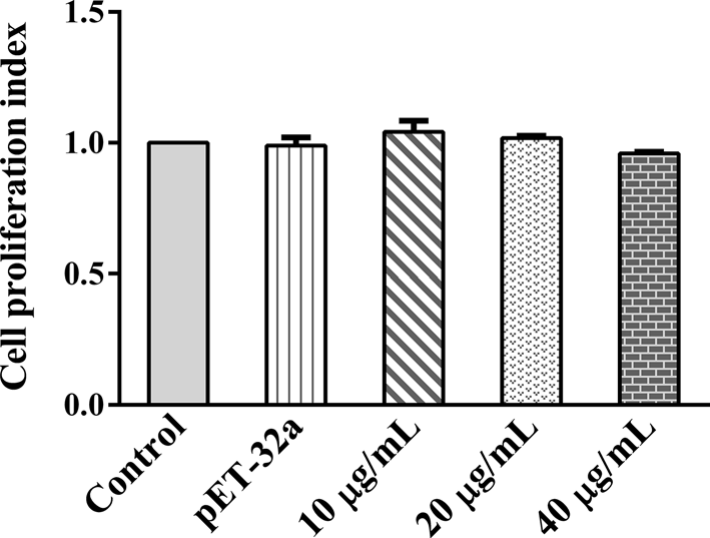
Influence of rHc-HAP on PBMC proliferation. PBMCs were treated with phosphate-buffered saline (negative control), purified pET-32a protein (positive control) and serial concentrations of rHc-HAP for 24 h. The cell proliferation index was determined by setting the OD_450_ values of the control group as 100%. Data are presented as the mean ± SEM from three independent experiments

The effect of rHc-HAP protein on PBMC proliferation was examined using a CCK-8 kit. The results showed that rHc-HAP protein did not affect the proliferation of goat PBMCs [pET-32a: ANOVA,* F*_(4, 10)_ = 1.591, *P* = 0.7556; 10 μg/ml: ANOVA,* F*_(4, 10)_  = 1.591, *P* = 0.2507; 20 μg/ml: ANOVA,* F*_(4, 10)_ = 1.591, *P* = 0.6182; 40 μg/ml: ANOVA,* F*_(4, 10)_ = 1.591, *P* = 0.2736] (Fig. 6).

### Effect of rHc-HAP protein on the apoptosis of goat PBMCs

**Fig. 7:**
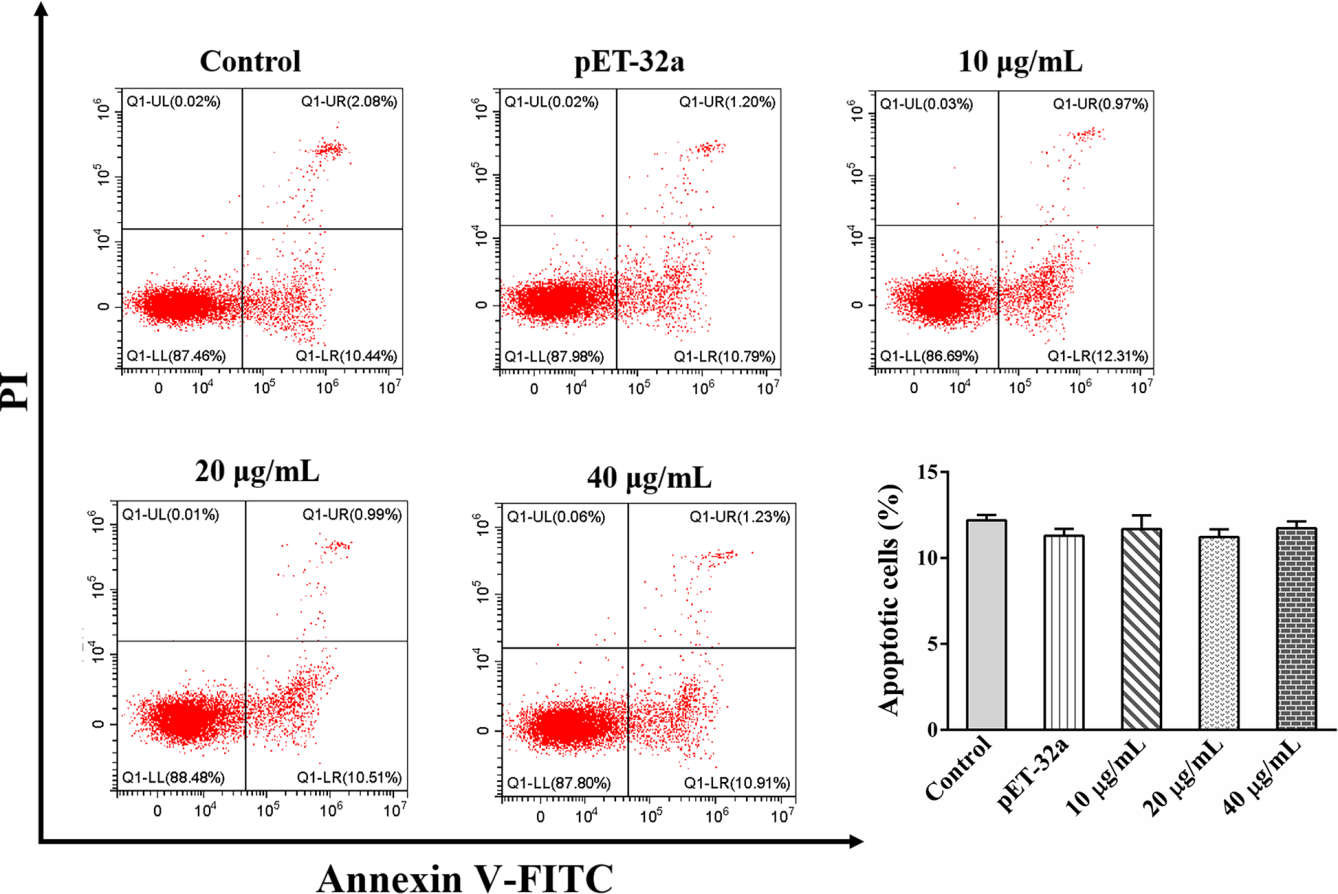
Effect of rHc-HAP on the apoptosis of goat PBMCs. Cells were incubated with serial concentrations of rHc-HAP and pET-32a for 24 h at 37 °C and 5% CO_2_. Data are presented as the mean ± SEM from three independent experiments

The effect of rHc-HAP protein on the apoptosis of PBMCs was examined by the Annexin V-FITC Apoptosis Detection Kit. The results showed that rHc-HAP protein did not affect the apoptosis of goat PBMCs [pET-32a: ANOVA,* F*_(4, 10)_ = 0.609, *P* = 0.2317; 10 μg/ml: ANOVA,* F*_(4, 10)_ = 0.609, *P* = 0.4777; 20 μg/ml: ANOVA,* F*_(4, 10)_ = 0.609, *P* = 0.1987; 40 μg/ml: ANOVA,* F*_(4, 10)_ = 0.609, *P* = 0.5198] (Fig. 7).

### Effect of rHc-HAP protein on NO secretion from goat PBMCs

**Fig. 8:**
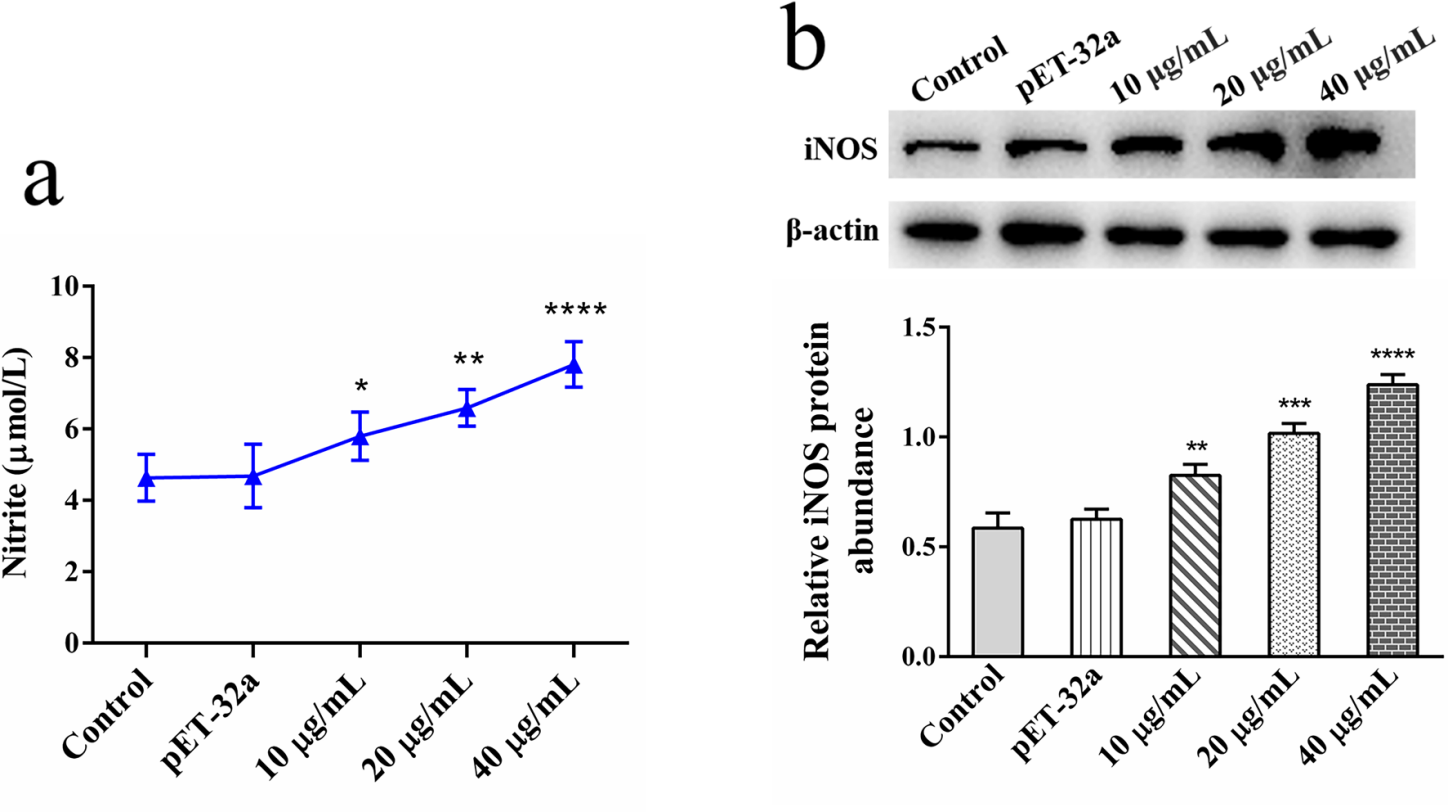
Effect of rHc-HAP on NO secretion from goat PBMCs. Cells were incubated with serial concentrations of rHc-HAP and pET-32a for 24 h at 37 °C and 5% CO_2_. **a** The effect of rHc-HAP on NO release from PBMCs was examined using a commercial kit. **b** The effect of rHc-HAP on the expression level of iNOS in PBMCs was analyzed using western blot assays. Data are presented as the mean ± SEM from three independent experiments. Asterisks indicate significant difference at *P < 0.05, **P < 0.01, ***P < 0.001, ****P < 0.0001 vs the control group. Abbreviations: iNOS, Iducible nitric oxide synthase; NO nitric oxide

As shown in Fig. 8, rHc-HAP protein increased nitrate levels in the cell supernatant in a dose dependent manner, indicating that rHc-HAP protein upregulates NO production in goat PBMCs [pET-32a: ANOVA,* F*_(4, 10)_ = 15.417, *P* = 0.9230; 10 μg/ml: ANOVA,* F*_(4, 10)_ = 15.417, *P* = 0.0298; 20 μg/ml: ANOVA,* F*_(4, 10)_ = 15.417, *P* = 0.0011; 40 μg/ml: ANOVA,* F*_(4, 10)_ = 15.417, *P* < 0.0001] (Fig. 8a). As expected, rHc-HAP protein promoted the expression of iNOS in goat PBMCs in a dose-dependent manner [pET-32a: ANOVA,* F*_(4, 10)_ = 27.770, *P* = 0.5905; 10 μg/ml: ANOVA,* F*_(4, 10)_ = 27.770, *P* = 0.0083; 20 μg/ml: ANOVA,* F*_(4, 10)_ = 27.770, *P* = 0.0002; 40 μg/ml: ANOVA,* F*_(4, 10)_ = 27.770, *P* < 0.0001] (Fig. 8b).

### rHc-HAP protein promoted immune responses in T helper cells in goat PBMCs

**Fig. 9:**
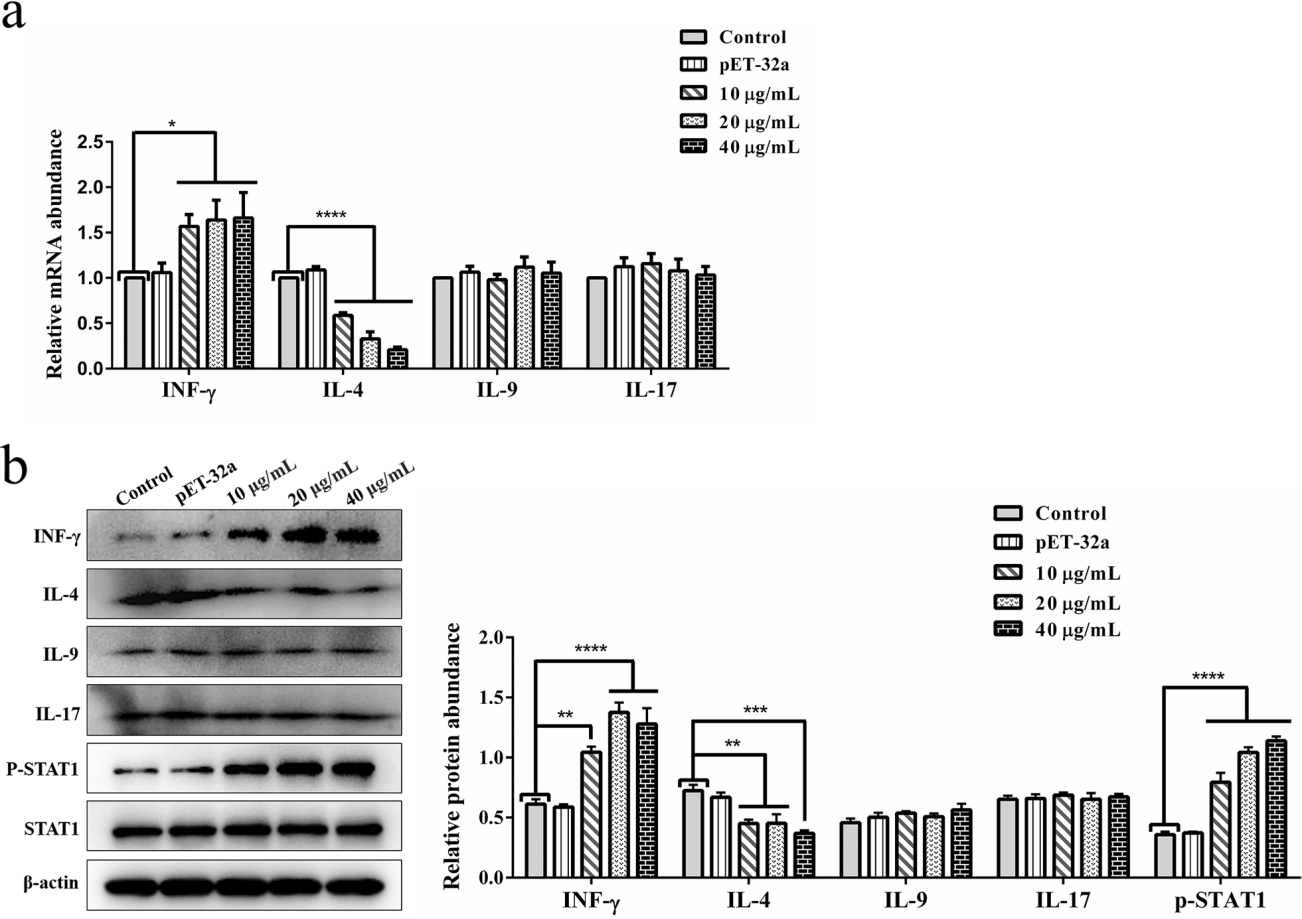
To assess the effect of rHc-HAP on the transcriptional expression of IFN-γ, IL-4, IL-9, IL-17 and p-STAT1 in goat PBMCs. **a** The transcript levels of IFN-γ, IL-4, IL-9, and IL-17 were detected by qPCR assays. **b** The expression levels of IFN-γ, IL-4, IL-9, IL-17, p-STAT1, and STAT1 were detected by western blot assays. Data are presented as the mean ± SEM from three independent experiments. Asterisks indicate significant differences at *P < 0.05, **P < 0.01, ***P < 0.001, ****P < 0.0001 vs the control group. Abbreviations: IFN, Interferon; IL, interleukin; p-STAT1, phosphorylated signal transducer and activator of transcription factor

The effect of rHc-HAP protein on the transcript levels of IFN-γ [t helper type 1 (Th1)], IL-4 (Th2), IL-9 (Th9) and IL-17 (Th17) in goat PBMCs was analyzed in qPCR assays. The results are shown in Fig. 9a. rHc-HAP protein dose-dependently upregulated the transcriptional abundance of IFN-γ [pET-32a: ANOVA,* F*_(4, 10)_ = 3.492, *P* = 0.8125; 10 μg/ml: ANOVA,* F*_(4, 10)_ = 3.492, *P* = 0.0448; 20 μg/ml: ANOVA,* F*_(4, 10)_ = 3.492, *P* = 0.0274; 40 μg/ml: ANOVA,* F*_(4, 10)_ = 3.492, *P* = 0.0234] in goat PBMCs and dose-dependently downregulated the transcriptional abundance of IL-4 [pET-32a: ANOVA,* F*_(4, 10)_ = 85.052, *P* = 0.1759; 10 μg/ml: ANOVA,* F*_(4, 10)_ = 85.052, *P* < 0.0001; 20 μg/ml: ANOVA,* F*_(4, 10)_ = 85.052, *P* < 0.0001; 40 μg/ml: ANOVA,* F*_(4, 10)_ = 85.052, *P* < 0.0001] in goat PBMCs. However, rHc-HAP protein did not affect the transcription of IL-9 [pET-32a: ANOVA,* F*_(4, 10)_ = 0.447, *P* = 0.5977; 10 μg/ml: ANOVA,* F*_(4, 10)_ = 0.447, *P* = 0.8713; 20 μg/ml: ANOVA,* F*_(4, 10)_ = 0.447, *P* = 0.3283; 40 μg/ml: ANOVA,* F*_(4, 10)_ = 0.447, *P* = 0.6646] and IL-17 [pET-32a: ANOVA,* F*_(4, 10)_ = 0.436, *P* = 0.3884; 10 μg/ml: ANOVA,* F*_(4, 10)_ = 0.436, *P* = 0.2796; 20 μg/ml: ANOVA,* F*_(4, 10)_ = 0.436, *P* = 0.5787; 40 μg/ml: ANOVA,* F*_(4, 10)_ = 0.436, *P* = 0.8156] in goat PBMCs. We also analyzed the effect of rHc-HAP protein on the expression levels of IFN-γ, IL-4, IL-9, IL-17, STAT1 and p-STAT1 in goat PBMCs by western blot assays. The results are shown in Fig. 9b. rHc-HAP protein dose-dependently promoted the expression of IFN-γ [pET-32a: ANOVA,* F*_(4, 10)_ = 24.013, *P* = 0.8274; 10 μg/ml: ANOVA,* F*_(4, 10)_ = 24.013, *P* = 0.0022; 20 μg/ml: ANOVA,* F*_(4, 10)_ = 24.013, *P* < 0.0001; 40 μg/ml: ANOVA,* F*_(4, 10)_ = 24.013, *P* < 0.0001] and p-STAT1 [pET-32a: ANOVA,* F*_(4, 10)_ = 65.669, *P* = 0.8063; 10 μg/ml: ANOVA,* F*_(4, 10)_ = 65.669, *P* < 0.0001; 20 μg/ml: ANOVA,* F*_(4, 10)_ = 65.669, *P* < 0.0001; 40 μg/ml: ANOVA,* F*_(4, 10)_ = 65.669, *P* < 0.0001], which indicated that rHc-HAP protein activated the IFN-γ/STAT1 signaling pathway in goat PBMCs. Consistent with the qPCR results, rHc-HAP protein dose-dependently inhibited IL-4 [pET-32a: ANOVA,* F*_(4, 10)_ = 10.425, *P* = 0.4348; 10 μg/ml: ANOVA,* F*_(4, 10)_ = 10.425, *P* = 0.0023; 20 μg/ml: ANOVA,* F*_(4, 10)_ = 10.425, *P* = 0.0024; 40 μg/ml: ANOVA,* F*_(4, 10)_ = 10.425, *P* = 0.0004] expression in goat PBMCs. However, rHc-HAP protein had no significant effect on the expression of IL-9 [pET-32a: ANOVA,* F*_(4, 10)_ = 1.281, *P* = 0.3957; 10 μg/ml: ANOVA,* F*_(4, 10)_ = 1.281, *P* = 0.1385; 20 μg/ml: ANOVA,* F*_(4, 10)_ = 1.281, *P* = 0.3424; 40 μg/ml: ANOVA,* F*_(4, 10)_ = 1.281, *P* = 0.0601], IL-17 [pET-32a: ANOVA,* F*_(4, 10)_ = 0.198, *P* = 0.8814; 10 μg/ml: ANOVA,* F*_(4, 10)_ = 0.198, *P* = 0.4789; 20 μg/ml: ANOVA,* F*_(4, 10)_ = 0.198, *P* = 0.9921; 40 μg/ml: ANOVA,* F*_(4, 10)_ = 0.198, *P* = 0.6766] and STAT1 in goat PBMCs. These results suggest that rHc-HAP protein upregulated the Th1 immune response in PBMCs while downregulating the Th2 immune response in PBMCs.

## Discussion

 The nematode *H. contortus,* which is considered to be one of the most common parasites infesting ruminant grazers, represents a major threat to the health of livestock herds [25, 26]. Currently, Barbervax® (Wormvax Australia Pty Ltd., Albany, WA, Australia) is the only vaccine that is commercially available in Australia. This vaccine uses natural H11 and H-gal-GP proteins of adult worms as antigens [27–29]. However, Barbervax® vaccine antigen extraction is relatively expensive, so there is a need to explore new vaccine targets. As nematode antigens are complex and immune-modulatory, vaccine development is challenging, and a comprehensive study of how the host immune system is influenced by *H. contortus* antigens is warranted. A transcript level of Hc-HAP has been found in the egg, L3, xL3 and adult stages of *H. contortus*, with the highest level of transcription in the L3 stage, suggesting that Hc-HAP may play an important role in the development of L3. However, the role of Hc-HAP in L3 development and environmental resistance remains to be demonstrated by subsequent studies. Interestingly, an aspartyl protease inhibitor protein previously identified in our laboratory from *H. contortus* has a similar transcriptional pattern to that of Hc-HAP and also affects the function of PBMCs [30]. In addition, the immunofluorescence results revealed that Hc-HAP was mainly expressed on the microvilli of *H. contortus*, which showed similar immunolocalization patterns to H11 and H-gal-GP antigens [31]. Theoretically, Hc-HAP can induce circulating antibodies, and these antibodies enter the parasite's intestine via the blood-sucking route and attack native Hc-HAP in the intestinal microvilli, thus potentially affecting the development and reproduction of the *H. contortus*. Interestingly, *Caenorhabditis elegans* pho-1 (a homolog of HAP, necessary for the development of the adult intestine) was also found mainly in the microvilli, consistent with immunolocalization data of Hc-HAP [16]. Based on these results, Hc-HAP may be a candidate for vaccine or drug development.

A major function of PBMCs is to present antigens to the host and assist with the host's innate defense against foreign pathogens [32, 33]. In previous studies, our group has shown that many antigenic molecules of *H. contortus* bind to PBMCs and affect their function [34–38]. The IFA results showed that rHc-HAP could bind to goat PBMCs, which may be a mechanism by which rHc-HAP could regulate the function of PBMCs. In addition, rHc-HAP could be recognized by sera from goats infected with *H. contortus*, suggesting that rHc-HAP may also serve as a diagnostic antigen for haemonchosis.

As a result of immune cells producing NO, the host is more likely to resist infection by parasites, bacteria and other pathogens [39–42]. Our study showed that rHc-HAP promoted the release of NO from PBMCs in a dose-dependent manner, which may be one of the defense mechanisms of the host to prevent parasite invasion.

It is important to note that in the fight against infection by pathogens such as parasites, Th1 (with its primary cytokine, IFN-γ), Th2 (with its main cytokine, IL-4), Th9 (with its main cytokine, IL-9) and Th17 (with its main cytokine, IL-17) immune responses play a vital role [43–47]. This study showed that rHc-HAP stimulated the IFN-γ/STAT1 pathway and promoted Th1-polarization of PBMCs, while suppressing Th2-polarization. IFN-γ activates STAT1, which phosphorylates in the nucleus, provoking an inflammatory response and activating M1 macrophages [48, 49]. By enzymatically promoting the expression of iNOS, M1 macrophages stimulate the release of pro-inflammatory cytokines, including NO [50]. This is consistent with the finding that rHc-HAP stimulates NO release from PBMCs. Thus, it might be hypothesized that rHc-HAP enhances NO release from PBMCs by activating the IFN-γ/STAT1 pathway.

However, the present study only characterized the transcription of Hc-HAP in different life stages of the *H. contortus* and its expression in adults. Future studies should focus on investigating the effects of rHc-HAP gene silencing or specific anti-Hc-HAP antibodies to block native Hc-HAP proteins during the growth and development of *H. contortus*. Additionally, it is necessary to further evaluate the protective effects of subunit or DNA vaccines utilizing Hc-HAP as an antigen in goats infected with *H. contortus*.

## Conclusions

The results of this study showed that Hc-HAP was transcribed in egg, L3, xL3, and adult (female/male) stages of *H. contortus* and was highly expressed at the intestinal microvilli of adult worms. Moreover, rHc-HAP promoted the polarization of PBMCs toward the Th1 immune response while inhibited the Th2 immune response.

## Supplementary Information

**Additional file 1: Table S1.** Primers used to amplify the Hc-HAP gene. **Table S2.** Primers used for qPCR experiments.

**Additional file 2: Figure S1.** Signal peptide prediction. The amino acid sequences of Hc-HAP (NCBI accession numbers: CDJ80664.1) were used to predict Signal peptides by SignalP 5.0 Server. There were no Signal peptides predicted in this protein structure. http://www.cbs.dtu.dk/services/SignalP/. **Figure S2.** Transmembrane structure prediction using TMHMM Server v.2.0. The amino acid sequences of Hc-HAP (NCBI accession numbers: CDJ80664.1) was analyzed to predict transmembrane structures using TMHMM Server v.2.0. There were no transmembrane domains was predicted in this protein structure. http://www.cbs.dtu.dk/services/TMHMM/.

## Data Availability

All data generated or analyzed during this study are included within the article and its file.

## Abbreviations

CCK-8: Cell Counting Kit-8
HAP: Histidine acid phosphatase
IFA: Immunofluorescence assay
IL: Interleukin
IFN-γ: Interferon gamma
IPTG: Isopropyl-β-D-thiogalactopyranoside
Ni–NTA: Ni^2+^-nitrilotriacetic acid
NO: Nitric oxide
qPCR: Quantitative real-time PCR
PBMCs: Peripheral blood mononuclear cells
PBS: Phosphate-buffered saline
PVDF: Polyvinyl difluoride
STAT: Signal transducer and activator of transcription factor
TBST: Tris-buffered saline containing 0.05% Tween-20
Th: CD4+ T helper cells
