# Supplementary material for: Histidine acid phosphatase domain-containing protein from Haemonchus contortus is a stimulatory antigen for the Th1 immune response of goat PBMCs
Source: Parasit Vectors. 2022 Aug 6;15:282. doi: 10.1186/s13071-022-05411-7 (PMC9356432; doi:10.1186/s13071-022-05411-7)
Supplement: Supplementary file 1 — Additional file 1: Table S1. Primers used to amplify the Hc-HAP gene. Table S2. Primers used for qPCR experiments. [file 13071_2022_5411_MOESM1_ESM.docx]

**Table S1. Primers used for PCR Amplification experiments**

| **Target genes** | **Primer sequences (5’-3’)** |
| --- | --- |
| HAP | CG**GGATCC**ATGCTTGGTATGTATGGTCAAGAAAAC |
|  | CC**CTCGAG**TCAGCAGCACATCCATAGAGCG |

Note: The underlined sequences are the *Bam* HI and *Hind* III digestion sites.

**Table S2. Primers used for qPCR experiments**

| **Target genes** | **Primer sequences (5’-3’)** |
| --- | --- |
| β-actin | CACCACACCTTCTACAAC |
|  | TCTGGGTCATCTTCTCAC |
| IL-4  IL-9 | GTACCAGCCACTTCGTCCAT |
|  | GCTGCTGAGATTCCTGTCAA  GATGCGGCTGATTGTTT  CTCGTGCTCACTGTGGAGT |
| IL-17 | TTGTAAAGGCAGGGGTCATC |
|  | GGTGGAGCGCTTGTGATAAT |
| IFN-γ | GAACGGCAGCTCTGAGAAAC |
|  | GGTTAGATTTTGGCGACAGG |
| Hc-HAP | CCAGACCAACCAATGAGCCAGTG |
|  | CAGCAGCACATCCATAGAGCGATC |
| β-tubulin | TGCTATGTTCCGTGGTCGTATG |
|  | CGGCAGTCTTAACGTTGTTTGG |
