# Supplementary material for: Histidine acid phosphatase domain-containing protein from Haemonchus contortus is a stimulatory antigen for the Th1 immune response of goat PBMCs
Source: Parasit Vectors. 2022 Aug 6;15:282. doi: 10.1186/s13071-022-05411-7 (PMC9356432; doi:10.1186/s13071-022-05411-7)
Supplement: Supplementary file 2 — Additional file 2: Figure S1. Signal peptide prediction. The amino acid sequences of Hc-HAP (NCBI accession numbers: CDJ80664.1) were used to predict Signal peptides by SignalP 5.0 Server. There were no Signal peptides predicted in this protein structure. http://www.cbs.dtu.dk/services/SignalP/. Figure S2. Transmembrane structure prediction using TMHMM Server v.2.0. The amino acid sequences of Hc-HAP (NCBI accession numbers: CDJ80664.1) was analyzed to predict transmembrane structures using TMHMM Server v.2.0. There were no transmembrane domains was predicted in this protein structure. http://www.cbs.dtu.dk/services/TMHMM/. [file 13071_2022_5411_MOESM2_ESM.docx]

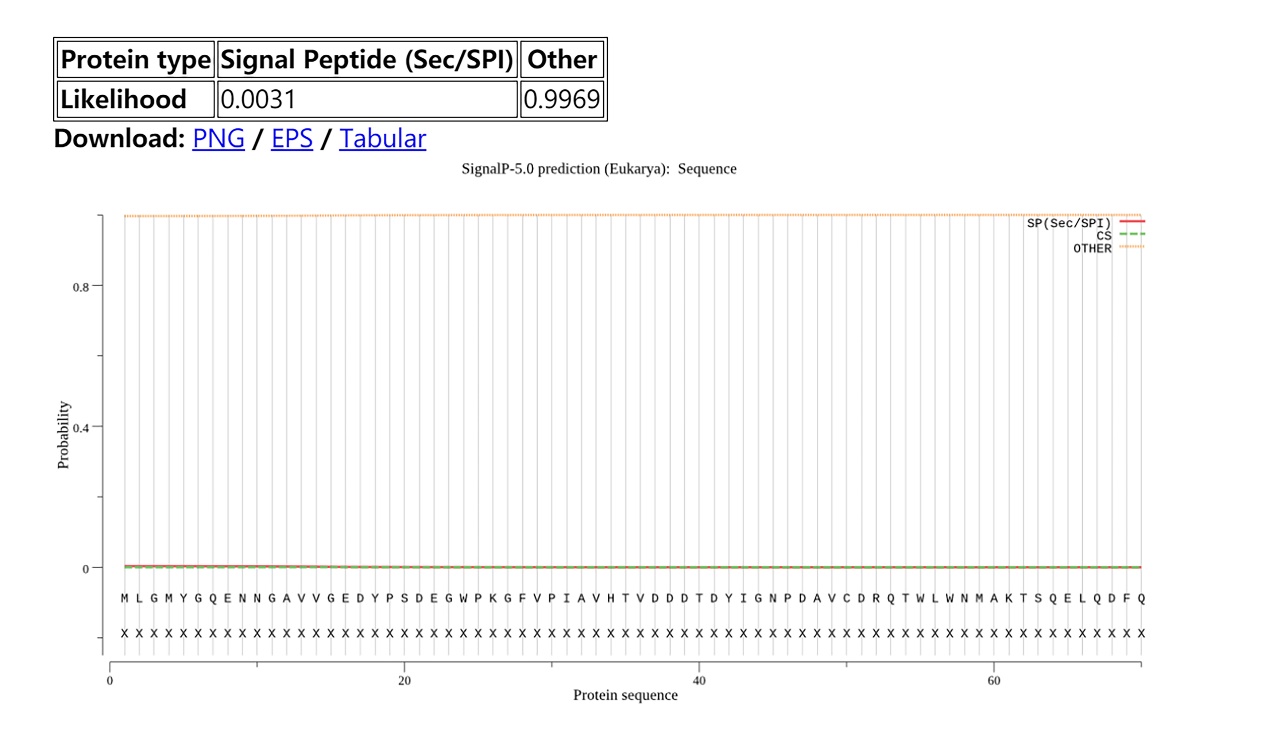


**Figure S1. Signal peptide prediction.** The amino acid sequences of Hc-HAP (NCBI

accession numbers: CDJ80664.1) was used to predict Signal peptides by SignalP 5.0 Server. There were no Signal peptides was predicted in this protein structure. http://www.cbs.dtu.dk/services/SignalP/.


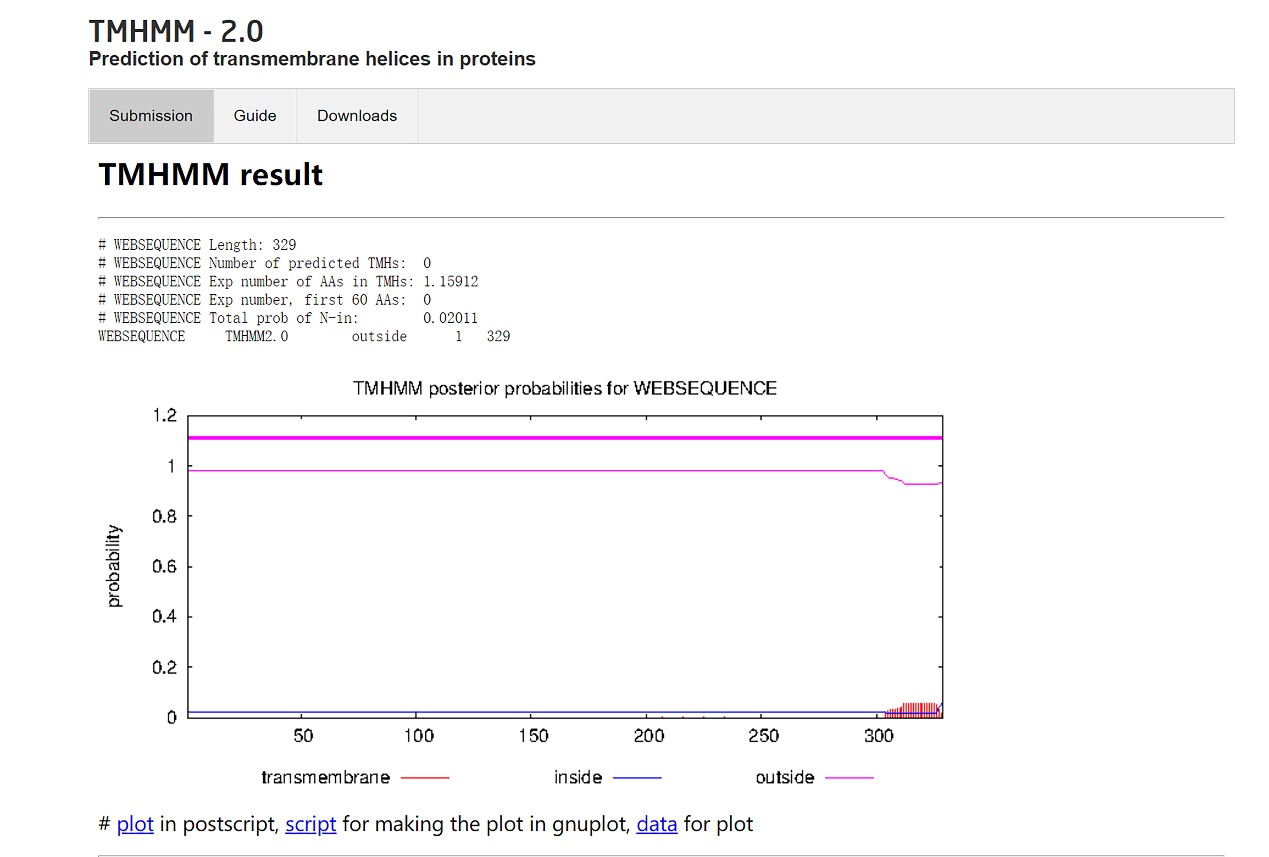


**Figure S2. Transmembrane structure prediction using TMHMM Server v.2.0.** The amino acid sequences of Hc-HAP (NCBI accession numbers: CDJ80664.1) was analyzed to predict transmembrane structures using TMHMM Server v.2.0. There were no transmembrane domains was predicted in this protein structure. <http://www.cbs.dtu.dk/services/TMHMM/>.
